# Multi-target mechanism of Naoshuantong capsule for treatment of Ischemic stroke based on network pharmacology and molecular docking

**DOI:** 10.1097/MD.0000000000035771

**Published:** 2023-11-03

**Authors:** Fengjiao Yang, Ya Yan, Yun Gu, Kezhen Qi, Jianjie Chen, Guangming Wang

**Affiliations:** a College of Pharmacy, Dali University, Dali, PR China; b School of Clinical Medicine, Dali University, Dali, PR China.

**Keywords:** IS, molecular docking, network pharmacology, NST capsules, Pharmacological mechanism

## Abstract

**Background:**

Naoshuantong capsule (NST capsule) is a classic Chinese patent medicine, which can treat ischemic stroke (IS) and has good clinical efficacy. However, its pharmacological mechanism remains to be further explored in the treatment of IS.

**Methods:**

The bio-active components and potential targets of NST Capsules were obtained by ETCM and TCMSP databases. In addition, the related targets of IS were collected by Genecard, OMIM, DrugBank, TTD and DisGeNET databases. NST-IS common target was obtained by Venn platform. PPI network of NST-IS common target and the composition - target network diagram of NST Capsule were constructed by Cytoscape3.8.1. Finally, AutoDock was used for molecular docking.

**Results:**

265 targets were predicted from 32 active compounds in NST Capsule, 109 common targets were identified between NST Capsule and IS. The top 10 key targets of PPI network were ALB, TNF, TP53, VEGFA, CASP3, MYC, etc. Enrichment analysis showed that NST capsules treated IS mainly through lipid and atherosclerosis, fluid shear stress and atherosclerosis signaling pathways.

**Conclusion:**

Through the methods of network pharmacology and molecular docking, this study clarified that NST capsules play a role in the treatment of IS, which is multi-target, multi-channel and multi-component regulation. This study further explored the pharmacological mechanism of NST capsule in the treatment of IS, which can provide some references for the subsequent research in the pharmacological mechanism of NST capsule.

## 1. Introduction

Stroke is one of the major diseases that cause death and disability in the world,^[1]^ it not only seriously threatens people’s health, but also causes a huge economic burden to society.^[2,3]^ The World Health Organization reports that about 5.5 million people die from stroke each year, of which Ischemic stroke (IS) accounts for 87%.^[4]^ IS refers to a disease of focal neurological deficit in the brain, which is caused by the interruption of cerebral blood flow caused by thrombosis or embolism in a certain area.^[5–7]^ It has the characteristics of high morbidity, high mortality and high disability rate.^[8,9]^ The occurrence of IS is closely related to several risk factors, including diabetes, smoking, hyperlipidemia and hypertension.^[10–13]^ After the occurrence of IS, the main manifestations are facial paralysis, crooked mouth, slurred speech, paralysis^[14]^ and other clinical symptoms. At present, Tissue plasminogen activator (r-tPA) is the main drug for the treatment of IS,^[15,16]^ but its use has a strict time limit and needs to be administered within 4.5 hours after the onset of IS, there is a risk of secondary brain damage during treatment and increases the risk of disability and stroke recurrence.^[17–19]^ It is necessary to explore new methods to prevent the occurrence of IS and treat the brain damage caused by IS.

In recent years, people pay more and more attention to traditional Chinese medicine (TCM) because of its low toxicity and good effect.^[20]^ Studies have shown that TCM has a good effect on IS, which can effectively prevent the occurrence of IS and delay the progress of the disease.^[21,22]^ Therefore, exploring the mechanism of action of TCM in the treatment of diseases will help to make better use of TCM in the treatment of diseases. Naoshuantong capsule (NST capsule) is a classic drug for the treatment of IS, which has been widely used in clinical practice.^[23]^ It is composed of Typha angustifolia L, Paeoniae Radix Rubra, Curcumae Radix, Rhizom Gastrodiae and Rhaponticum uniflorum (L.) DC.^[24]^ It has the effects of promoting blood circulation, eliminating wind and phlegm, improving blood circulation and protecting nerve cells, which is consistent with the pathogenesis of IS.^[25]^ NST capsule can effectively reduce the brain damage caused by IS.^[26]^ However, the mechanism of NST capsule in the treatment of IS is not very clear, Therefore, it is very important to systematically study the multi-component, multi-target and multi-pathway mechanism of NST capsule in the treatment of IS.

**Figure 1.**
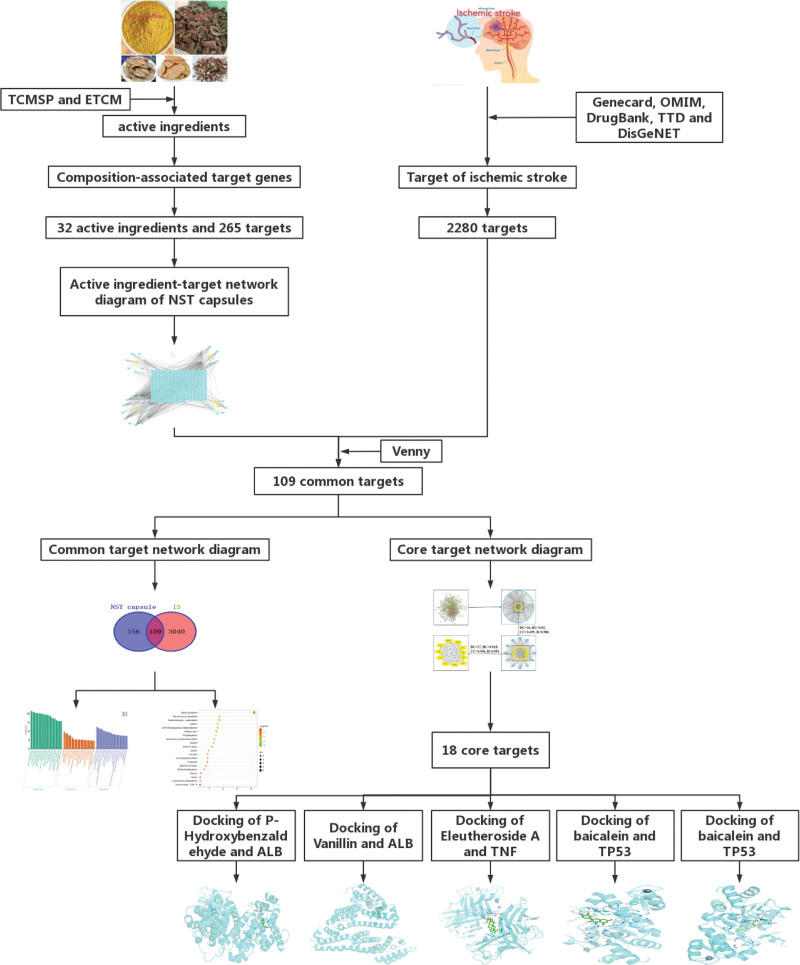
Flow chart of this study.

TCM compound preparations are often composed of a variety of Chinese herbs, which have many components that it is difficult to elucidate their mechanism of action by traditional methods. Network pharmacology can reveal the mechanism of action of TCM prescriptions in treating diseases by constructing a multi-layer network of drug-component-gene-disease, which is consistent with the holistic view of TCM,^[27]^ it provide a method to study the mechanism of action of complex components of TCM.^[28]^ In this study, we explored the targets and pathways of NST capsule in the treatment of IS by network pharmacology and molecular docking methods, then further elucidated the action mechanism of NST capsule in the treatment of IS. The research workflow is shown in Figure 1. This study can provide some scientific basis for the treatment of IS with NST capsule, which can promote the rational application of NST capsule.

## 2. Materials and methods

### 2.1. Collection of targets of NST Capsule and IS

The main components of NST capsule are Typha angustifolia L, Paeoniae Radix Rubra, Curcumae Radix, Rhizom Gastrodiae and Rhaponticum uniflorum (L.) DC Based on the network pharmacological method, the active ingredient and related targets of Typha angustifolia L, Paeoniae Radix Rubra, Curcumae Radix, Rhizom Gastrodiae and Rhaponticum uniflorum (L.) DC are collected through Traditional Chinese Medicine Systems Pharmacology, Database and Analysis Platform (TCMSP; http://tcmspw.com/TCMSP.PHP) database^[29]^ and ETCM database (http://www.tcmip.cn/ETCM/index.php/Home/Index/),^[30]^ if OB > 30% and DL > 0.18^[31]^ are set, the active components of NST capsule can be obtained, its related targets can be obtained according to its active components. The obtained relevant targets were converted into standard gene names by Uniprot platform (https://www.uniprot.org).^[29]^ “Cerebral arterial thrombosis” was set as the search term, then IS related to target is collected by Genecard database (https://www.genecards.org/),^[32]^ OMIM database (https://omim.org/),^[33]^ providing database (http://db.idrblab.net/ TDD/)^[34]^ and DisGeNET database (http://www.disgenet.org/),^[35]^ The obtained genes were pooled and duplicated genes were further deleted, finally, the final gene set of IS was obtained. NST capsule gene set and IS set to be imported Venny2.1.0 platform (https://bioinfogp.cnb.csic.es/tools/venny/)^[36]^ to gain common genes of NST-IS.

### 2.2. Construction of active ingredient-target network diagram of NST capsule

In order to construct the “active ingredient-target” network diagram of NST Capsule, the active compounds and targets of the 5 ingredients of NST Capsule were imported into Cytoscape3.8.1, then the corresponding attribute files were imported into Cytoscape3.8.1, the “active ingredient-target” network diagram of NST Capsule were obtained. Finally, The component-target network diagram was refined by using Style in Cytoscape3.8.1. In the network diagram, nodes represent 5 TCM, active compounds and targets, edges represent the relationship between nodes.

### 2.3. Construction of PPI network diagram and key sub-network diagram

The Protein-Protein Interaction (PPI) network diagram of the common target of NST capsule and IS was constructed based on the String platform (https://string-db.org/),^[37]^ the common target of NST capsule and IS was imported into the String platform, medium reliability was set as 0.400, the PPI network data table was derived. The PPI data table was imported into Cytoscape3.8.1 to calculate the obtained network diagram and export the calculated data table, then the median of degree values (D), betweenness centrality (BC), closeness centrality (CC), and R were calculated, Degree above 2 times the median, BC, CC and R above the median are set, the sub-network diagram was obtained. D, BC, CC, and R medians are calculated again in the data table. D, BC, CC, and R above the median are set, finally, the core network diagram was obtained.

### 2.4. Common target enrichment analysis of NST capsule and IS

Based on the common genes of NST capsules and IS, the common gene sets of NST capsules and IS were performed for gene ontology (GO) and Kyoto Encyclopedia of Genes and Genomes (KEGG) enrichment analysis by the metascape platform (http://www.metascape.org/),^[38]^ which can explore the potential mechanism of action of NST capsules in the treatment of IS. GO enrichment analysis includes cell component (CC), molecular function (MF) and biological process (BP) analysis, KEGG enrichment analysis includes key signaling pathway analysis.

### 2.5. Molecular docking of key targets

The 3 most significant genes were selected in the core target network diagram for molecular docking. The corresponding receptor proteins of the 3 target were obtained from the Uniprot database, the structure of the proteins was obtained from the PDB database (http://www.rcsb.org/).^[39]^ ChemDraw18.1 software was used to draw the chemical structure of the corresponding components of the 3 genes. In order to obtain the 3D structure of the components, after energy was minimized, the 3D structure of each component was exported by Chem3D 18.1 software. PyMOL software was used to remove the water molecules of the receptor proteins and components, the receptor proteins were imported into Autodock software for hydro-treating and charge calculation. Based on Autodock Vina software, docking pockets and docking parameters were set to perform molecular docking between receptor proteins and small molecular ligands.

## 3. Results

### 3.1. Active compounds and potential targets of NST Capsule and IS

**Figure 2.**
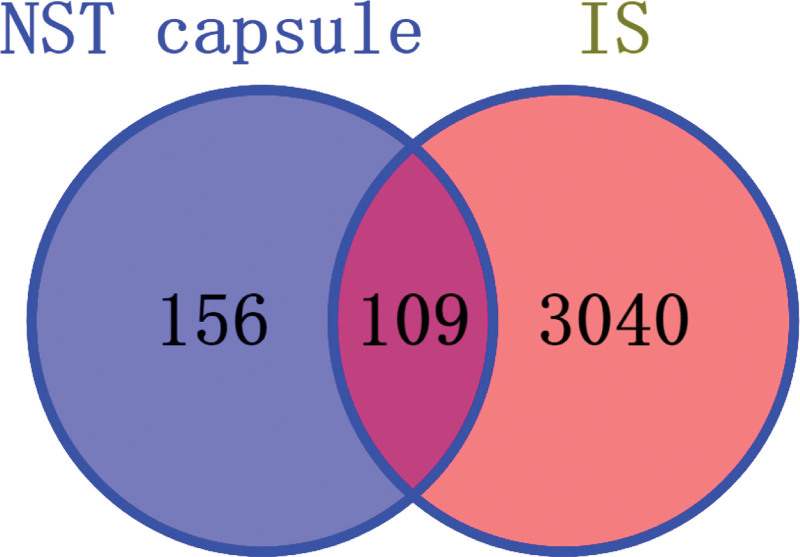
Venn diagram of drug and disease common targets.

According to ETCM database, NST capsule contains Typha angustifolia L, Paeoniae Radix Rubra, Curcumae Radix, Rhizom Gastrodiae and Rhaponticum uniflorum (L.) DC. Based on TCMSP database, there were 8 active compounds and 153 targets in Typha angustifolia L, 29 active compounds and 61 targets in Paeoniae Radix Rubra, 15 active compounds and 29 targets in Curcumae Radix, 5 components and 21 targets in Rhaponticum uniflorum (L.) DC. 21 components and 291 targets of Rhizom Gastrodiae were obtained from ETCM database. 78 active compounds and 555 related targets were obtained from the TCMSP database and ETCM database. After 46 common components and 290 common targets were removed, 32 components and 265 targets were obtained. Based on Genecard database, OMIM database, TTD database and DisGeNET database, 3041, 129, 0 and 15 IS-related targets were obtained respectively, then 36 common targets were removed, finally, 3149 IS-related targets were obtained. Based on Venn platform, a total of 109 targets of NST capsule in the treatment of IS were predicted. The Venn diagram is shown in Figure 2.

### 3.2. Analysis of active components-target network diagram of NST capsule

**Figure 3.**
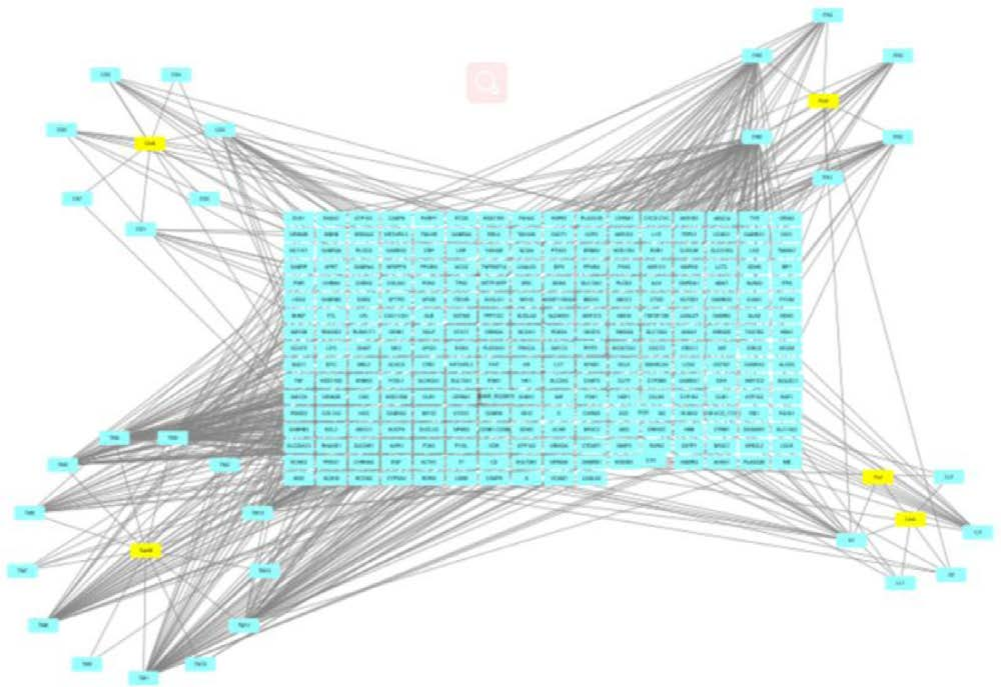
“Active ingredient-target” network diagram of NST capsules. NST capsule = Naoshuantong capsule.

Based on Cytoscape3.8.1 software, active ingredients and targets of NST capsule were analyzed. 303 nodes and 559 edges were obtained. The composition and target network diagram of NST capsules are shown in Figure 3. In the network diagram, 5 nodes represent 5 TCM in NST capsule, 30 nodes represent the active components in NST capsule, 2 nodes represent the common active components in NST capsule, 266 nodes represent the targets of NST capsule, 559 edges represent the interaction relationship between components and targets. In the network diagram, Rhizom Gastrodiae has the most active components and targets, which indicated that Rhizom Gastrodiae was the key component in NST capsule.

### 3.3. Core network diagram analysis of NST capsule and IS

**Figure 4.**
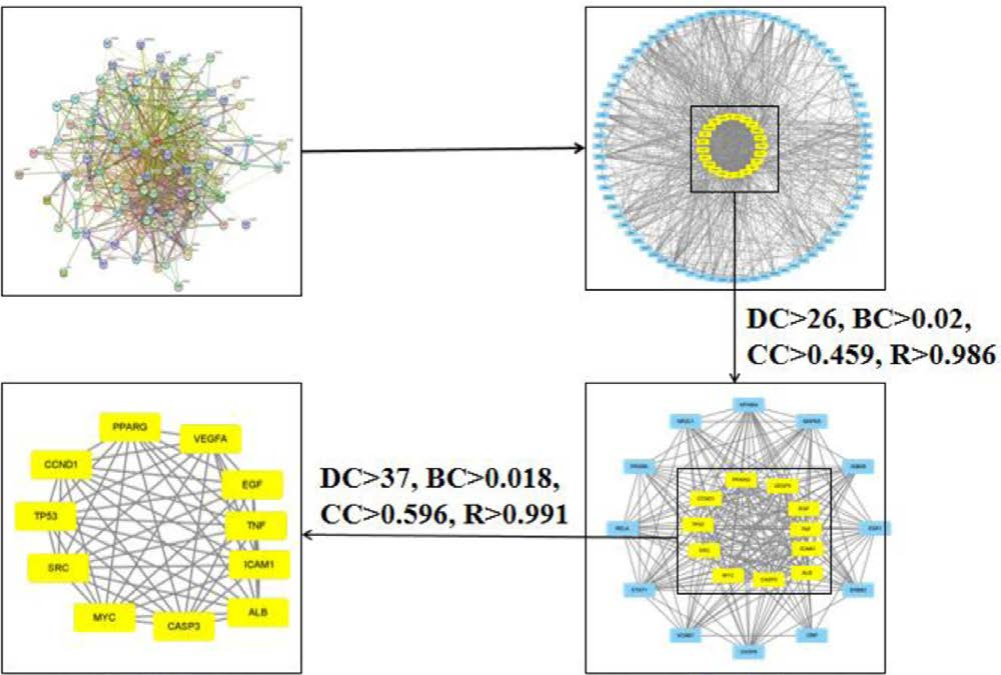
Core target screening flow chart of NST capsule for IS. IS = Ischemic stroke, NST capsule = Naoshuantong capsule.

Based on the String platform, the PPI network diagram of the common targets between NST capsule and IS was obtained. Based on Cytoscape3.8.1, PPI network diagrams were analyzed, the median double of Degree is 26, the BC median is 0.02, the CC median is 0.495, and the R median is 0.986. The above data were used to screen PPI networks, 23 core targets are obtained. Then, according to the filtered data table, Degree value 37, BC value 0.018, CC value 0.596, *R* value 0.991 can be calculated, the above data are used for secondary screening of PPI network, then 11 core targets are obtained. Among them, the degree values of ALB, TNF, and TP53 were the highest, which indicated that these 3 targets were the key targets of NST capsule in the treatment of IS. The core target screening process is shown in Figure 4.

### 3.4. GO and KEGG enrichment analysis of common targets of NST capsules and IS

**Figure 5.**
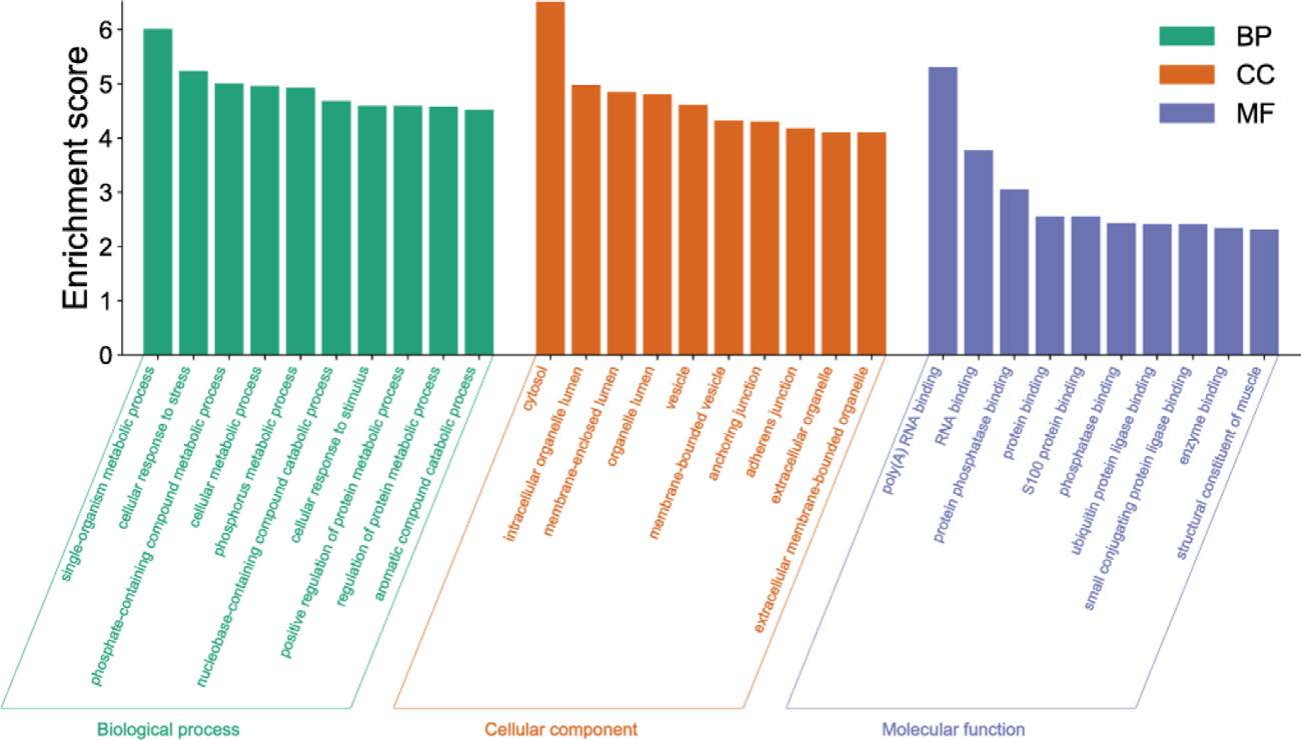
GO entry diagram. GO = gene ontology.

**Figure 6.**
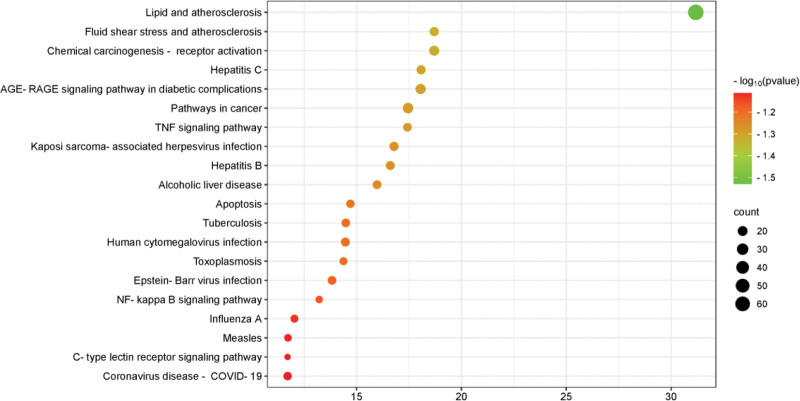
KEGG bubble diagram. KEGG = Kyoto Encyclopedia of Genes and Genomes.

Based on GO enrichment analysis, the GO pathway of the common target of NST capsule and IS can be obtained. In GO pathway analysis, 321 terms pathway were obtained from biological process (BP) analysis, 85 terms pathway were obtained from cell component (CC) analysis, 120 terms pathway were obtained from molecular function (MF) analysis. The 10 most significant GO terms pathway are shown in Table 1, the first 10 terms pathway of GO analysis are shown in Figure 5. BP analysis showed that the target was mainly enriched in response to foreign biological stimuli, hormones, nutrient levels, antioxidant, positive regulation of cell death, extracellular stimulation, hypoxia, oxygen content reduction, etc. CC analysis showed that the targets were mainly concentrated in cystic cavity, secretory granulosa cavity, membrane raft, membrane microregion, transcriptional regulatory complex, receptor complex, NMDA selective glutamate receptor complex, extracellular matrix, etc. From the perspective of MF, the targets are mainly concentrated in transcription factor binding, DNA binding transcription factor binding, nuclear receptor activity, ligand-activated transcription factor activity, RNA polymerase II specific DNA binding transcription factor binding, steroid-binding proteins, etc. KEGG enrichment analysis identified 181 pathways, which can suggest that these target play a role in the treatment of IS by influencing lipid and atherosclerosis, fluid shear stress and atherosclerosis pathways. In conclusion, the targets of the main active components of NST capsule are distributed in different metabolic pathways, which may be the mechanism of action of NST capsule in the treatment of IS. The 20 most significant KEGG pathways are shown in Table 1, the bubble diagram of their pathways is shown in Figure 6.

**Table 1:** GO functional enrichment analysis and KEGGpathway analysis.

Category	Serial number	Description	LogP	Log(q-value)	Gene
Cellular components	GO:0031983	Vesicle lumen	−9.789473282	−6.514	ALAD, ALB, ALOX5, APOB, CTSD, EGF, FTL, IDH1, LCN2, MIF, NFKB1, RNASE3, VEGFA, SELP
	GO:0034774	Secretory granule lumen	−8.750269342	−5.907	ALAD, ALB, ALOX5, CTSD, EGF, FTL, IDH1, LCN2, MIF, NFKB1, RNASE3, VEGFA
	GO:0060205	Cytoplasmic vesicle lumen	−8.704904671	−5.907	ALAD, ALB, ALOX5, CTSD, EGF, FTL, IDH1, LCN2, MIF, NFKB1, RNASE3, VEGFA
	GO:0045121	Membrane raft	−7.655178688	−5.065	CASP3, CASP8, CTSD, HK1, IKBKB, OLR1, SELE, SLC2A4, SRC, TNF, TNFRSF1A
	GO:0098857	Membrane microdomain	−7.64144997	−5.065	CASP3, CASP8, CTSD, HK1, IKBKB, OLR1, SELE, SLC2A4, SRC, TNF, TNFRSF1A
	GO:0005667	Transcription regulator complex	−6.513983137	−4.017	PARP1, CCND1, RUNX2, ESR1, MYC, NFE2L2, PPARG, RELA, STAT1, TP53, VDR, NR1I2
	GO:0043235	Receptor complex	−5.492265889	−3.092	ERBB2, GRIN1, GRIN2A, IKBKB, LDLR, NR3C2, OLR1, PPARG, TNFRSF1A, VDR, GRIN3A, CALM1, CASP3, CASP8, KCNK2, PRKCA, VCAM1, CHRM3, SRC
	GO:0017146	NMDA selective glutamate receptor complex	−5.445783704	−3.092	GRIN1, GRIN2A, GRIN3A
	GO:0101002	Ficolin-1-rich granule	−5.345499508	−3.092	ALAD, ALOX5, CTSD, HBB, IDH1, LGALS3, MIF, OLR1
	GO:0031012	Extracellular matrix	−5.333025903	−3.092	ACHE, ANXA1, COL1A2, COL3A1, CTSD, F7, ICAM1, LGALS3, MMP3, PRSS1, VEGFA, ALB, APOB, P4HA2
Cellular components	GO:0031983	Vesicle lumen	−9.789473282	−6.514	ALAD, ALB, ALOX5, APOB, CTSD, EGF, FTL, IDH1, LCN2, MIF, NFKB1, RNASE3, VEGFA, SELP
	GO:0034774	Secretory granule lumen	−8.750269342	−5.907	ALAD, ALB, ALOX5, CTSD, EGF, FTL, IDH1, LCN2, MIF, NFKB1, RNASE3, VEGFA
	GO:0060205	Cytoplasmic vesicle lumen	−8.704904671	−5.907	ALAD, ALB, ALOX5, CTSD, EGF, FTL, IDH1, LCN2, MIF, NFKB1, RNASE3, VEGFA
	GO:0045121	Membrane raft	−7.655178688	−5.065	CASP3, CASP8, CTSD, HK1, IKBKB, OLR1, SELE, SLC2A4, SRC, TNF, TNFRSF1A
	GO:0098857	Membrane microdomain	−7.64144997	−5.065	CASP3, CASP8, CTSD, HK1, IKBKB, OLR1, SELE, SLC2A4, SRC, TNF, TNFRSF1A
	GO:0005667	Transcription regulator complex	−6.513983137	−4.017	PARP1, CCND1, RUNX2, ESR1, MYC, NFE2L2, PPARG, RELA, STAT1, TP53, VDR, NR1I2
	GO:0043235	Receptor complex	−5.492265889	−3.092	ERBB2, GRIN1, GRIN2A, IKBKB, LDLR, NR3C2, OLR1, PPARG, TNFRSF1A, VDR, GRIN3A, CALM1, CASP3, CASP8, KCNK2, PRKCA, VCAM1, CHRM3, SRC
	GO:0017146	NMDA selective glutamate receptor complex	−5.445783704	−3.092	GRIN1, GRIN2A, GRIN3A
	GO:0101002	Ficolin-1-rich granule	−5.345499508	−3.092	ALAD, ALOX5, CTSD, HBB, IDH1, LGALS3, MIF, OLR1
	GO:0031012	Extracellular matrix	−5.333025903	−3.092	ACHE, ANXA1, COL1A2, COL3A1, CTSD, F7, ICAM1, LGALS3, MMP3, PRSS1, VEGFA, ALB, APOB, P4HA2
Molecular functions	GO:0008134	Transcription factor binding	−12.43569709	−8.740	PARP1, BCL2, RUNX2, ESR1, HSF1, HSPB1, MYC, NFE2L2, NFKBIA, PGR, PPARA, PPARG, RELA, RORA, SRC, STAT1, TP53, VDR, NR1I2
	GO:0140297	DNA-binding transcription factor binding	−11.930686	−8.536	PARP1, BCL2, RUNX2, ESR1, HSF1, HSPB1, MYC, NFE2L2, NFKBIA, PPARA, PPARG, RELA, SRC, STAT1, TP53, VDR, NR1I2
	GO:0004879	Nuclear receptor activity	−11.35057571	−8.257	ESR1, ESR2, NR3C1, PPARA, PPARG, RORA, VDR, NR1I2, APOD, CYP3A4, NR3C2, PGR, MYC, NFE2L2, RELA, STAT1
	GO:0098531	Ligand-activated transcription factor activity	−11.35057571	−8.257	ESR1, ESR2, NR3C1, PPARA, PPARG, RORA, VDR, NR1I2
	GO:0061629	RNA polymerase II-specific DNA-binding transcription factor binding	−10.58228454	−7.586	PARP1, ESR1, HSF1, HSPB1, NFE2L2, NFKBIA, PPARA, PPARG, RELA, SRC, STAT1, TP53, VDR, NR1I2
	GO:0005496	Steroid binding	−10.07902593	−7.161	APOD, CYP3A4, ESR1, ESR2, NR3C1, NR3C2, PGR, RORA, VDR
	GO:0003707	Nuclear steroid receptor activity	−9.657343605	−6.836	ESR1, ESR2, NR3C1, NR3C2, PGR, PPARA
	GO:0001221	Transcription coregulator binding	−9.629034351	−6.836	ESR1, MYC, NFE2L2, PGR, PPARA, PPARG, RELA, RORA, STAT1
	GO:0031406	Carboxylic acid binding	−9.267206155	−6.526	ALB, GRIN1, PLA2G1B, PLOD1, PPARG, SELE, SELP, VDR, P4HA2, GRIN3A, HBA1, HBB
	GO:0016491	Oxidoreductase activity	−9.082785518	−6.387	ALDH2, AKR1B1, ALOX5, CYP1B1, CYP2B6, CYP3A4, NQO1, HBA1, HBB, IDH1, IDH2, PLOD1, PTGS1, TYR, VCAM1, P4HA2, ABCC4, FTL, LCN2
KEGG Pathway	hsa05417	Lipid and atherosclerosis	−33.72512442	−31.179	APOB, BCL2, CALM1, CASP3, CASP8, CYP2B6, ICAM1, IFNB1, IKBKB, LBP, LDLR, MMP3, NFE2L2, NFKB1, NFKBIA, OLR1, PPARG, PRKCA, MAPK8, RELA, SELE, SELP, SRC, TNF, TNFRSF1A, TP53, VCAM1, NQO1, GSTM1, VEGFA, CCND1, EGF, MYC, PPARA, STAT1, YWHAE, BIRC5, ERBB2, ESR1, ESR2, IRF1, ACACA, ALDH2, PARP1, CTSD, VDR, ALOX5, TNFSF13B, PRSS1, C1R, MBL2, HSPB1, RASA1, COL1A2, LCN2, SLC2A4, ABCC1, CYP1B1, HK1, ABCC4, CHRM3, GRIN1, GRIN2A, LPL, HSF1, RORA, RUNX2
	hsa05418	Fluid shear stress and atherosclerosis	−20.94936087	−18.704	BCL2, CALM1, NQO1, GSTM1, ICAM1, IKBKB, NFE2L2, NFKB1, MAPK8, RELA, SELE, SRC, TNF, TNFRSF1A, TP53, VCAM1, VEGFA
	hsa05207	Chemical carcinogenesis – receptor activation	−20.77123955	−18.702	BIRC5, CCND1, BCL2, CYP1B1, CYP2B6, CYP3A4, EGF, ESR1, ESR2, GSTM1, MYC, NFKB1, PGR, PPARA, PRKCA, RELA, SRC, VDR, VEGFA, CALM1, CTSD
	hsa05160	Hepatitis C	−20.01975561	−18.075	CCND1, CASP3, CASP8, EGF, IFNB1, IKBKB, LDLR, MYC, NFKB1, NFKBIA, PPARA, RELA, STAT1, TNF, TNFRSF1A, TP53, YWHAE
	hsa04933	AGE-RAGE signaling pathway in diabetic complications	−19.90288089	−18.055	CCND1, BCL2, CASP3, COL1A2, COL3A1, ICAM1, NFKB1, PRKCA, MAPK8, RELA, SELE, STAT1, TNF, VCAM1, VEGFA, NFKBIA, SRC, PARP1, CTSD, PPARA, SLC2A4, HSPB1
	hsa05200	Pathways in cancer	−19.22359935	−17.455	BIRC5, CCND1, BCL2, CALM1, CASP3, CASP8, NQO1, EGF, ERBB2, ESR1, ESR2, GSTM1, IKBKB, MYC, NFE2L2, NFKB1, NFKBIA, PPARG, PRKCA, MAPK8, RELA, STAT1, TP53, VEGFA
	hsa04668	TNF signaling pathway	−19.12571371	−17.424	CASP3, CASP8, ICAM1, IFNB1, IKBKB, IRF1, MMP3, NFKB1, NFKBIA, MAPK8, RELA, SELE, TNF, TNFRSF1A, VCAM1
	hsa05167	Kaposi sarcoma-associated herpesvirus infection	−18.42627489	−16.783	CCND1, CALM1, CASP3, CASP8, ICAM1, IFNB1, IKBKB, MYC, NFKB1, NFKBIA, MAPK8, RELA, SRC, STAT1, TNFRSF1A, TP53, VEGFA
	hsa05161	Hepatitis B	−18.19796234	−16.606	BIRC5, BCL2, CASP3, CASP8, IFNB1, IKBKB, MYC, NFKB1, NFKBIA, PRKCA, MAPK8, RELA, SRC, STAT1, TNF, TP53
	hsa04936	Alcoholic liver disease	−17.52374884	−15.977	ACACA, ALDH2, CCND1, CASP3, CASP8, IFNB1, IKBKB, LBP, NFKB1, NFKBIA, PPARA, MAPK8, RELA, TNF, TNFRSF1A
	hsa04210	Apoptosis	−16.21414852	−14.709	PARP1, BIRC5, BCL2, CASP3, CASP8, CTSD, IKBKB, NFKB1, NFKBIA, MAPK8, RELA, TNF, TNFRSF1A, TP53
	hsa05152	Tuberculosis	−15.95414445	−14.487	BCL2, CALM1, CASP3, CASP8, CTSD, IFNB1, LBP, NFKB1, MAPK8, RELA, SRC, STAT1, TNF, TNFRSF1A, VDR
	hsa05163	Human cytomegalovirus infection	−15.89802012	−14.465	CCND1, CALM1, CASP3, CASP8, IFNB1, IKBKB, MYC, NFKB1, NFKBIA, PRKCA, RELA, SRC, TNF, TNFRSF1A, TP53, VEGFA
	hsa05145	Toxoplasmosis	−15.77680006	−14.376	ALOX5, BCL2, CASP3, CASP8, IKBKB, LDLR, NFKB1, NFKBIA, MAPK8, RELA, STAT1, TNF, TNFRSF1A
	hsa05169	Epstein-Barr virus infection	−15.20133448	−13.831	CCND1, BCL2, CASP3, CASP8, ICAM1, IFNB1, IKBKB, MYC, NFKB1, NFKBIA, MAPK8, RELA, STAT1, TNF, TP53
	hsa04064	NF-kappa B signaling pathway	−14.56013006	−13.218	PARP1, BCL2, ICAM1, IKBKB, LBP, NFKB1, NFKBIA, RELA, TNF, TNFRSF1A, VCAM1, TNFSF13B
	hsa05164	Influenza A	−13.35080689	−12.035	CASP3, CASP8, ICAM1, IFNB1, IKBKB, NFKB1, NFKBIA, PRKCA, PRSS1, RELA, STAT1, TNF, TNFRSF1A
	hsa05162	Measles	−13.01843747	−11.727	CCND1, BCL2, CASP3, CASP8, IFNB1, IKBKB, NFKB1, NFKBIA, MAPK8, RELA, STAT1, TP53
	hsa05171	Coronavirus disease – COVID-19	−12.9603907	−11.708	C1R, IFNB1, IKBKB, MBL2, MMP3, NFKB1, NFKBIA, PRKCA, MAPK8, RELA, SELP, STAT1, TNF, TNFRSF1A
	hsa04625	C-type lectin receptor signaling pathway	−12.9537702	−11.708	CALM1, CASP8, IKBKB, IRF1, NFKB1, NFKBIA, MAPK8, RELA, SRC, STAT1, TNF

GO = gene ontology, KEGG = Kyoto Encyclopedia of Genes and Genomes.

### 3.5. Molecular docking analysis

**Figure 7.**
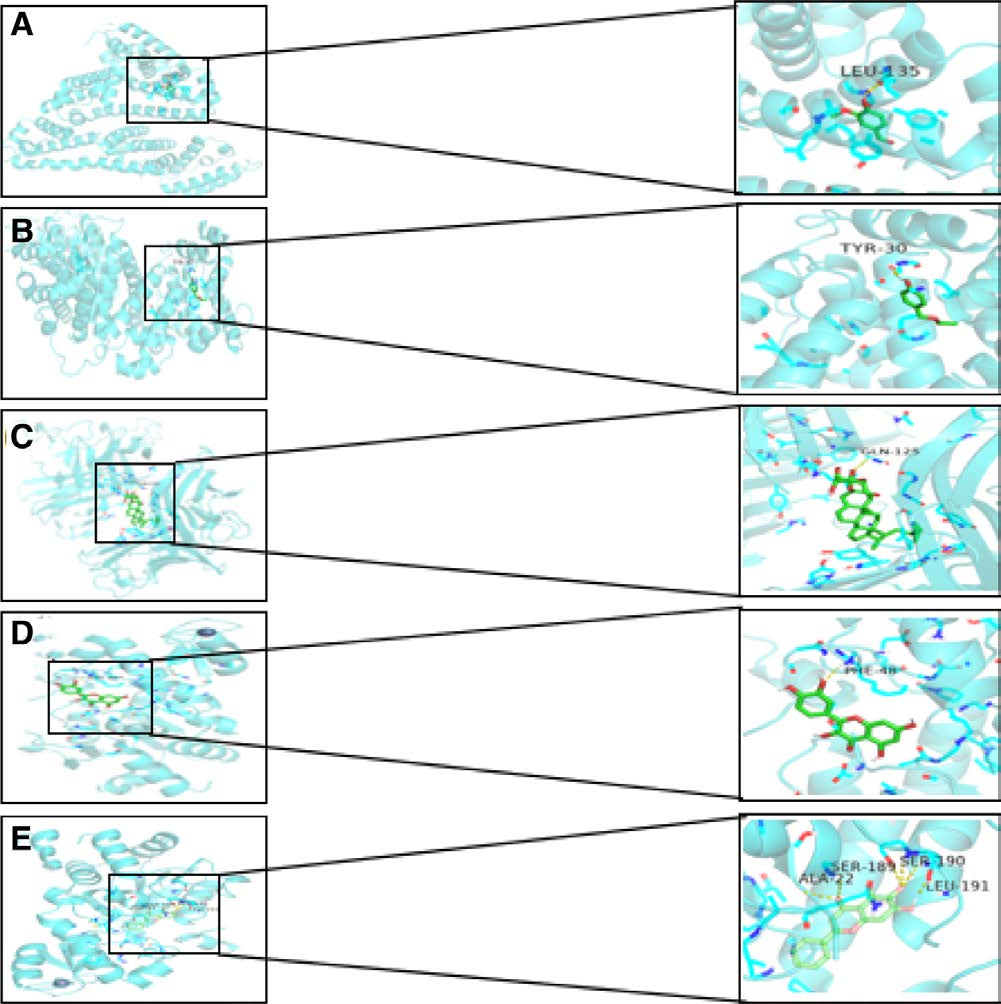
Three-dimensional schematic diagram of the molecular docking model and active site of the main components of P-Hydroxybenzaldehyde, Vanillin, Eleutheroside A, Baicalein and Quercetin. (A) Docking of P-Hydroxybenzaldehyde and ALB. (B) Docking of Vanillin and ALB. (C) Docking of Eleutheroside A and TNF. (D) Docking of baicalein and TP53. (E) Docking of Quercetin and TP53.

After the core target network diagram was analyzed, the top 3 targets ALB, TNF and TP53 were selected, their corresponding active ingredients were Vanillin, P-Hydroxybenzaldehyde, Eleutheroside A, Baicalin and Quercetin respectively. Molecular docking was performed between the target and its corresponding protein. After molecular docking, 5 docking results were obtained, the binding energy of all molecular docking results was less than −5.4 KJ, which indicate that 5 molecules could combine well with their targets and play a good role in the treatment of IS. The docking results are shown in Figure 7. The binding energy is shown in Table 2.

**Table 2:** Binding energy for molecular docking of core targets.

Composition-target	Binding energy (KJ)
Vanillin and ALB	−6.1
P-Hydroxybenzaldehyde and ALB	−6.5
Eleutheroside A and TNF	−9.1
Baicalein and TP53	−7.6
Quercetin and TP53	−8.9

## 4. Discussion

IS seriously affects people’s physical health and brings serious economic burden to patients and their families.^[40]^ The pathogenesis of IS is complex, its prevention and recovery require multiple-target and multiple-pathways to play a role at the same time.^[41]^ In recent years, NST Capsule has been widely used in the treatment of IS. The drug is composed of 5 TCM, such as Typha angustifolia L, Paeoniae Radix Rubra, Curcumae Radix, Rhizom Gastrodiae and Rhaponticum uniflorum (L.) DC. The drug contains a variety of effective components. However, the mechanism of NST capsule in the treatment of IS remains to be further explored. In this study, network pharmacology and molecular docking methods were used to further explore the pharmacological mechanism of NST capsule in the treatment of IS.

In this study, 2280 targets of IS were obtained by Genecard database, OMIM database, DrugBank database, TTD database and DisGeNET database. Based on TCMSP and ETCM databases, 32 effective components and 265 targets were obtained from Typha angustifolia L, Paeoniae Radix Rubra, Curcumae Radix, Rhizom Gastrodiae and Rhaponticum uniflorum (L.) DC. The NST-IS has 109 common targets. Through network pharmacological analysis, we found that the core targets of NST capsule in the treatment of IS include ALB, TNF, TP53, CASP3, VEGFA, SRC, MYC, PPARG, ICAM1, EGF, CCND1, etc. These targets have the effects of anti-oxidation, anti-apoptosis, anti-inflammation, regulation of blood lipids and atherosclerosis. Based on the core target network diagram, the top 5 components were selected: Vanillin, P-Hydroxybenzaldehyde, Eleutheroside A, Baicalein and Quercetin. Vanillin has the effect of antioxidant, anti-apoptotic, anti-inflammatory, neuroprotective and maintaining the integrity of the blood-brain barrier, which has a good effect in the treatment of IS.^[42]^ Experiments have shown that P-Hydroxybenzaldehyde can regulate the expression of Bcl-2, Bax and cleaved caspase-3 proteins, which can improve oxidative stress and mitochondrial dysfunction, so play a protective role in the injured brain.^[43]^ Eleutheroside A is mainly involved in tissue inflammation and autoimmune response, which is related to the recovery of IS. Baicalein can protect mitochondria and promote neuronal protective factors through antioxidant, anti-apoptotic, anti-inflammatory and anti-excitotoxic pathways, which are involved in the prevention and recovery of IS.^[44]^ Quercetin can inhibit the secretion of inflammatory cytokines by immune cells and reduce platelet aggregation, which can reduce the formation of inflammatory thrombosis and play a role in the treatment of IS.^[45]^ Rhizom Gastrodiae has more active ingredients in NST capsules, so it can be inferred that Rhizom Gastrodiae is an important component of NST capsules. According to reports, Rhizom Gastrodiae has the effect of anti-oxidative stress, reduces neuroinflammation and inhibits apoptosis. Therefore, Rhizom Gastrodiae plays a role in treating IS by protecting the blood-brain barrier. By analyzing the PPI network of NST capsule and IS, we found that the core targets of NST capsule in the treatment of IS include ALB, TNF, TP53, CASP3, VEGFA, SRC, etc. These targets are involved in transcriptional regulation, gene regulation, apoptosis regulation and other biological processes. These biological processes are closely related to the occurrence of IS.

Base on network pharmacology and molecular docking, ALB, TNF and TP53 are the key targets of NST Capsule in the treatment of IS. According to reports, ALB has a good effect on the treatment of recurrent IS, which mainly plays a role in the treatment of IS by enhancing the synthesis of neuroprotectin D1 and resisting the synthesis of lipoxins and inflammatory factors. In addition, ALB can also play an anti-coagulant role by inhibiting platelet aggregation and increasing the production of anti-aggregation prostaglandin D2, which can play a role in the treatment of IS.^[46]^ NST capsule can inhibit the expression of pro-inflammatory factor TNF, which suggest that NST capsule may alleviate brain injury of IS by regulating lipid and atherosclerosis, fluid shear stress and atherosclerotic pathways.^[47]^ According to literature reports, TP53 factor can play a role in regulating glycolysis and apoptosis of cytokines by transferring glucose metabolism to pentose phosphate metabolic pathway, which can reduce oxidative stress response of brain nerves and play a protective role in injured ischemic neurons.^[48]^ Through network pharmacology and molecular docking, we found that the mechanism of action of NST capsule in the treatment of IS is related to ALB, TNF, TP53 and other targets, At the same time, lipid and atherosclerosis, fluid shear stress, atherosclerosis and chemical carcinogenic-receptor activation pathways are important. Our results are consistent with published research.

This study elucidated the mechanism of NST capsule in the treatment of IS through network pharmacology and molecular docking, which will provide a more reliable basis for the clinical use of NST capsule in the treatment of IS, it can promote the rational use of NST capsule. Secondly, this study can provide some reference for the future research of NST capsule. However, this study only explored the theoretical part of NST capsule in the treatment of IS, this study did not conduct in vitro experiments to verify the theoretical results. In the future research, we hope that researchers will conduct deeper research on the mechanism of NST capsule.

## 5. Conclusion

In this study, based on network pharmacology and molecular docking, we designed a strategy to analyze the pharmacological mechanism of NST Capsule in the treatment of IS. We found that hormone, nutrient level, positive regulation of cell death, extracellular stimulation, oxygen content reduction and other processes are closely related to the treatment of IS with NST capsule, these processes are related to lipid and atherosclerosis, fluid shear stress and atherosclerosis pathways. This indicates that NST capsules play a role in the treatment of IS through multi-target and multi-pathway. This study provides basic evidence for the drug safety management of NST capsule in the treatment of IS, this can prove that it is feasible to analyze the pharmacological mechanism of NST capsule by using network pharmacological strategies, which is conducive to the systematic safety management and evaluation of NST capsules.

## Author contributions

**Formal analysis:** Kezhen Qi.

**Methodology:** Yun Gu.

**Resources:** Guangming Wang.

**Software:** Fengjiao Yang.

**Supervision:** Ya Yan.

**Validation:** Jianjie Chen.

**Visualization:** Yun Gu, Ya Yan.

**Writing – original draft:** Fengjiao Yang.

**Writing – review & editing:** Guangming Wang.
